# Nanotherapeutic Modulation of Human Neural Cells and Glioblastoma in Organoids and Monocultures

**DOI:** 10.3390/cells9112434

**Published:** 2020-11-07

**Authors:** Issan Zhang, Paula Lépine, Chanshuai Han, María Lacalle-Aurioles, Carol X.-Q. Chen, Rainer Haag, Thomas M. Durcan, Dusica Maysinger

**Affiliations:** 1 Department of Pharmacology and Therapeutics, McGill University, 3655 Promenade Sir-William-Osler, Montreal, QC H3G 1Y6, Canada; issan.zhang@mail.mcgill.ca; 2 The Neuro’s Early Drug Discovery Unit (EDDU), McGill University, 3801 University Street, Montreal, QC H3A 2B4, Canada; paula.lepine@mcgill.ca (P.L.); chanshuai.han2@mcgill.ca (C.H.); maria.lacalleaurioles@mcgill.ca (M.L.-A.); xiuqing.chen@mcgill.ca (C.X.-Q.C.); thomas.durcan@mcgill.ca (T.M.D.); 3 Institute of Chemistry and Biochemistry, Freie Universität Berlin, Takustraße 3, 14195 Berlin, Germany; haag@chemie.fu-berlin.de

**Keywords:** cerebral organoids, nanomedicines, astrocytes, microglia, glioblastoma, lipocalin-2, inflammation

## Abstract

Inflammatory processes in the brain are orchestrated by microglia and astrocytes in response to activators such as pathogen-associated molecular patterns, danger-associated molecular patterns and some nanostructures. Microglia are the primary immune responders in the brain and initiate responses amplified by astrocytes through intercellular signaling. Intercellular communication between neural cells can be studied in cerebral organoids, co-cultures or in vivo. We used human cerebral organoids and glioblastoma co-cultures to study glia modulation by dendritic polyglycerol sulfate (dPGS). dPGS is an extensively studied nanostructure with inherent anti-inflammatory properties. Under inflammatory conditions, lipocalin-2 levels in astrocytes are markedly increased and indirectly enhanced by soluble factors released from hyperactive microglia. dPGS is an effective anti-inflammatory modulator of these markers. Our results show that dPGS can enter neural cells in cerebral organoids and glial cells in monocultures in a time-dependent manner. dPGS markedly reduces lipocalin-2 abundance in the neural cells. Glioblastoma tumoroids of astrocytic origin respond to activated microglia with enhanced invasiveness, whereas conditioned media from dPGS-treated microglia reduce tumoroid invasiveness. Considering that many nanostructures have only been tested in cancer cells and rodent models, experiments in human 3D cerebral organoids and co-cultures are complementary in vitro models to evaluate nanotherapeutics in the pre-clinical setting. Thoroughly characterized organoids and standardized procedures for their preparation are prerequisites to gain information of translational value in nanomedicine. This study provides data for a well-characterized dendrimer (dPGS) that modulates the activation state of human microglia implicated in brain tumor invasiveness.

## 1. Introduction

The brain is particularly challenging to model because of its complex structure and functions. Neural cells constantly interact with each other through signaling molecules as well as cell–cell contacts [1,2,3]. Monocultures are useful to answer cell type-specific questions, but processes involving multiple cell types benefit from the use of co-cultures and organoid models [4,5,6].

Although primary dissociated and organotypic slice cultures are attractive models to investigate molecular mechanisms and functions, particularly for genetic knock-ins and knock-outs, key differences between human and mouse brains can impact the translational potential of experimental results [7,8]. Primary human neural cells are valuable resources, but their use is often restrained by access or methodological limitations. Advances in stem cell research have led to the development of organoids as in vitro models resembling human tissues in structure and complexity [4,5,6]. Cerebral organoids differentiated from induced pluripotent stem cells (iPSCs) show organized neurons and astrocytes in three-dimensions (3D) [9,10].

Nanostructures have been extensively studied in different cell lines and rodents [11], but a limited amount of data are available in human primary cells and organoids. Rodent models were valuable for the assessment of absorption, distribution, elimination and metabolism of nanomedicines. They clearly showed problems related to the entry of some nanostructures into the cerebral parenchyma due to the blood–brain barrier [12,13]. Studies in rodents also provided information on how nanostructures can be tuned in terms of size, shape and surface properties to facilitate brain entry. Similar studies are clearly not possible in humans and are rare in non-human primate models (e.g., Onpattro, Abraxane, BIND-014) [14,15,16]. Organoids therefore offer a valuable platform for the testing of organic and metallic nanostructures.

Among the nanostructures that have been studied in rodents, which showed pronounced intrinsic anti-inflammatory properties, is dendritic polyglycerol sulfate (dPGS) [17,18,19,20,21]. Their structures with terminal sulfate groups resemble that of heparan sulfate, which exerts anti-coagulant effects [17,22,23,24]. In contrast to heparan sulfate, dPGS shows primarily anti-inflammatory activity with relatively weak anti-coagulant effects that are size- and charge-dependent. Earlier studies indicated that dPGS can effectively reduce hyperactivity of microglia stimulated by danger-associated molecular patterns and lipopolysaccharide (LPS) in mice [18,19,25]. Microglia are the resident immune cells of the central nervous system and constantly survey their surroundings under physiological conditions [26,27,28]. Their morphology is altered and their phagocytic functions are elevated under many pathological conditions, making them attractive targets for nanotherapeutic interventions. Our previous studies showed that dPGS can modulate microglial activation in response to pathogen-associated molecular patterns and misfolded proteins (e.g., amyloid beta), thereby reducing losses in dendritic spine density of excitatory hippocampal neurons in mouse organotypic slice cultures [18,19,25]. These studies also showed that hyperactive microglia generate reactive astrocytes through the release of acute-phase cytokines such as interleukin-6, tumor necrosis factor alpha and lipocalin-2 (LCN2). However, the abundance and release of LCN2 in human neural cells has not yet been reported. We tested if dPGS can modulate LCN2 in a human cerebral organoid model.

Aside from establishing human cerebral organoids, we also used glioblastoma tumoroids to investigate the effectiveness of nanotherapeutics in the brain tumor microenvironment. Glioblastomas are mainly astrocytomas characterized by their infiltrative nature [29]. Microglia and normal astrocytes in the glioblastoma environment promote disease progression by secreting soluble factors (e.g., cytokines and growth factors) [30,31,32]. By combining reconstituted 3D models of glioblastoma (tumoroids) with organoids and modulating the activity of microglia with dPGS, we demonstrate that dPGS could be a powerful therapeutic agent that can reduce inflammatory markers and glioblastoma invasiveness.

## 2. Materials and Methods

### 2.1. Generation of Cerebral Organoids from Human iPSC

Procedures for culturing human iPSCs, embedding organoids in optimal cutting temperature (OCT) blocks, cryosectioning and immunostaining were all described previously [10]. The cell-line used was the NCRM1 iPSC line obtained from the National Institutes of Health. The method used to generate the 3D cerebral organoids was adapted from the protocol published by Lancaster and Knoblich [9]. Compositions of the different media are the same and reagents used are similar, except for: DMEM-F12 (Gibco, Ottawa, ON, Canada), human embryonic stem cell (hESC) quality fetal bovine serum (FBS) (Wisent, St-Bruno, QC, Canada), MEM Non-Essential Amino Acids Solution (MEM-NEAA) (Multicell, Montreal, QC, Canada), 2-mercaptoethanol (Millipore Sigma, Oakville, ON, Canada), Y27632 Rho-associated protein kinase (ROCK) inhibitor (Selleckchem, Burlington, ON, Canada), N2 supplement (Gibco) and Penicillin-Streptomycin (Multicell).

For the generation and maintenance of embryoid bodies (EBs, days 0–11), we started from a 100 mm dish with iPSCs at 70% confluence with high quality (less than 10% differentiated cells). Cells were washed with DMEM-F12 and dissociated with Accutase to generate a suspension of single cells. Cells were gently resuspended in hESC media containing ROCK inhibitor [9], basic fibroblast growth factor (FGF-b) and plated at a density of 10,000 cells/well in a 96-well ultra-low attachment U-bottomed plate (Corning, Burlington, ON, Canada). Plates were centrifuged at 1200 rpm for 10 min and incubated at 37 °C, 5% CO_2_ for 48 h. During day 2, a half media change was made, followed by a media change every other day up to day 11. Images of EBs were recorded with Evos XL Core Microscope (Thermo Fisher Scientific, Ottawa, ON, Canada) every 2 days during media changes to measure their diameter using the Image J software (U. S. National Institutes of Health, Bethesda, Maryland, USA). Once EBs had reached ≥ 350 µm in diameter, hESC media was switched to be without ROCK inhibitor and FGF-b [9]. When EBs were about 500–600 µm in diameter with smooth and bright edges, the media was switched to neuronal induction media (NIM). EBs were maintained in NIM for 4–5 days until the outer surface was optically translucent and ready to be embedded in Matrigel^®^ (Corning) containing reduced growth factor droplets.

Matrigel^®^ was thawed on ice. After media removal from each well containing the EBs, 30 µL of Matrigel^®^ was added per well and incubated for 30 min at 37 °C. Next, 200 µL of final differentiation media without vitamin A [9] was added per well. Using a 1000 µL cut tip, the Matrigel^®^-embedded organoids were transferred into an uncoated 6-well ultra-low attachment plate (Corning) with 4 mL of final differentiation media without vitamin A per well. A maximum of five organoids were added and maintained per well to avoid fusion. Following transfer, plates were left for 48 h in stationary culture. After 48 h, media was changed to fresh final differentiation media without vitamin A, followed by a second 48 h incubation. Following the second media change, plates were transferred onto an orbital shaker set at 70 rpm (Scientific Industries Inc., Bohemia, NY, USA) housed in a 37 °C incubator. Media was changed every 3 days with fresh final differentiation media with vitamin A until the organoids were ready for analysis.

### 2.2. Immunofluorescence (IF) Staining in Organoids

Cerebral organoids were fixed with 4% paraformaldehyde (overnight at 4 °C), washed in phosphate-buffered saline (PBS, Multicell), dehydrated in 20% sucrose (1–3 days) and embedded in OCT Compound (Thermo Fisher Scientific). Blocks were kept at −80 °C until needed. For IF, samples were sectioned (20 μm) in a cryostat (Cryostar NX70, Thermo Fisher Scientific), air dried at room temperature (RT) and kept at −20 °C before IF staining. Cryosections were rehydrated in PBS (15 min) and permeabilized/blocked in blocking solution (1 h at room temperature, RT) containing: 0.2% Triton X-100 (Millipore Sigma), 0.05% bovine serum albumin and 5% normal donkey serum in PBS, before incubation (overnight at 4 °C) with primary antibodies. Antibodies used were as follows: mouse anti-Nestin (1:250, Developmental Studies Hybridoma Bank, Iowa City, IA, USA, rat-401), rabbit anti-SOX2 (1:500, Millipore, Oakville, ON, Canada, AB5603), chicken anti-MAP2 (1:1000, EnCor Biotechnology, Gainesville, FL, USA, CPCA-MAP2), rat anti-CTIP2 (1:500, Abcam, Toronto, ON, Canada, ab18465), rabbit anti-GFAP (1:250 Millipore, MAB144P), chicken anti-Beta III Tubulin (1:400, Millipore, AB9354), rabbit anti-Nestin (1:200, Abcam, ab92391) and rat anti-lipocalin-2 (1:500, R&D Systems, Toronto, ON, Canada, MAB1757). Sections were then washed in PBS (45 min) and incubated (1 h at RT) with secondary antibodies in blocking solution: goat anti-rat Dylight 488 (1:300, Abcam, ab96887), donkey anti-rabbit Dylight 594 (1:400, Abcam, ab96877), donkey anti-chicken Alexa 647 (1:500, Invitrogen, Ottawa, ON, Canada, A21447), donkey anti-mouse Dylight 550 (1:200, Abcam, ab96876), goat anti-chicken Dylight 650 (1:200, Abcam, ab96950), donkey anti-rabbit Dylight 550 (1:200, Abcam, ab96892) and goat anti-rat Alexa Fluor 647 (1:500, Thermo Fisher Scientific, A21247). Sections were washed (45 min) with PBS and incubated (10 min at RT) with Hoechst 33342 (1:5000, Thermo Fischer Scientific) in PBS, washed in PBS (10 min) and mounted using Aqua-Poly/Mount (Polysciences Inc., Warrington, PA, USA). Samples were imaged on a Leica TCS SP8 confocal microscope (Leica, Richmond Hill, ON, Canada) at a 20× magnification.

### 2.3. Cell Culture

U251N human glioblastoma cells and HMC3 human microglia were originally obtained from the American Type Culture Collection. Unless otherwise indicated, cells were maintained in Dulbecco’s Modified Eagle Medium (DMEM, Thermo Fisher Scientific) supplemented with 5% fetal bovine serum (FBS, Wisent) and 1% Penicillin-Streptomycin (Thermo Fisher Scientific). Primary human astrocytes were obtained from Dr. Jack Antel’s lab and maintained in DMEM supplemented with 5% FBS and 1% Penicillin-Streptomycin. Primary astrocytes were kept at passages below five. Conditioned media was obtained from cell cultures maintained in the exponential growth phase and used fresh at 50% (*v*/*v*) in fresh media following centrifugation (3,000 rpm, 5 min) to remove cell debris. When used for the treatment of cerebral organoids, conditioned media was prepared using organoid media.

### 2.4. Time-Dependent dPG/dPGS Internalization in Organoids and Tumoroids

Human cerebral organoids were treated with dPG-Cy5 (1 μM) or dPGS-Cy5 (1 μM) for 1 h, 4 h or 24 h, then used for IF staining or direct imaging. For direct imaging, organoids were fixed in 4% paraformaldehyde overnight and nuclei were labeled with Hoechst 33342 (10 μM, overnight). Organoids were washed in PBS and imaged using a fluorescence microscope. Fluorescence was analyzed in ImageJ. Glioblastoma tumoroids were treated with dPG-Cy5 (1 μM) or dPGS-Cy5 (1 μM) for 24 h, then washed twice with PBS before imaging using a fluorescence microscope.

### 2.5. Western Blot

Western blot analysis followed published procedures [33]. In brief, organoids were washed twice in cold PBS, then were cut into small pieces using a razor blade and incubated in RIPA lysis buffer for 30 min on ice. Monolayer cells were washed twice with cold PBS, then incubated in RIPA lysis buffer for 30 min on ice. Lysates were vortexed for 10 sec every 10 min and lastly centrifuged for 30 min at 4 °C and 13,000 rpm. Lysates and media samples were separated by SDS-PAGE and blotted onto PVDF membranes (Bio-Rad, Mississauga, ON, Canada). Blocked membranes were probed with primary antibodies: rat anti-lipocalin-2 (1:500, R&D Systems, MAB1757), rabbit anti-NFκB p65 (1:1000, Abcam, ab16502), mouse anti-STAT3 (1:1000, Abcam, ab119352), mouse anti-β-actin (1:5000, Millipore Sigma, A5316) or mouse anti-alpha-tubulin (1:5000, Abcam, ab7291) overnight at 4 °C. Membranes were washed and incubated with secondary antibodies (goat anti-rat HRP, 1:1000, Thermo Fisher Scientific, 31470, goat anti-rabbit HRP, 1:1000, Bio-Rad, 1706515 and horse anti-mouse HRP, 1:5000, Cell Signaling, Burlington, ON, Canada, 7076S) for 1 h at RT. Membranes were washed and incubated with enhanced chemiluminescence substrate (Bio-Rad) for 5 min, signals were acquired with an Amersham 6000 imager (Amersham, Oakville, ON, Canada) or on film, and quantified in ImageJ.

### 2.6. Collagen Invasion Assay

U251N tumoroids and human astrocyte spheroids were prepared using the hanging drop method [34]. Briefly, drops of 5,000 cells in 30 µL medium were pipetted onto the inner side of a 100 mm Petri dish (Thermo Fisher Scientific) lid. The lid was quickly flipped to cover the Petri dish filled with 20 mL PBS. Hanging drops were cultured at 37 °C for 48 h to allow tumoroids and spheroids to form. Tumoroids and spheroids were then gently scooped into a medium-filled Petri dish coated with 2% agarose (dissolved in PBS) and cultured for 48 h. Tumoroids and spheroids were implanted in collagen gel (Advanced BioMatrix, San Diego, CA, USA), in the presence or absence of primary human astrocytes (7,000 cells) and human HMC3 microglia (3,000 cells) dispersed in 200 µL gel. Gels were covered with 200 µL DMEM with or without treatment. Tumoroids and spheroids were imaged using light microscopy immediately after implantation (time = 0 day) and after 6 days. The average distance of cell outgrowth into the surrounding collagen was measured in ImageJ. For invasion assays of tumoroids into organoids, fluorescently-labeled tumoroids were prepared using U251N cells incubated with CellTracker Red (Thermo Fisher Scientific) following recommendations from the manufacturer. Tumoroids were placed in culture with organoids and observed to adhere after 24 h. Cultures were imaged using a fluorescence microscope over 6 days.

### 2.7. Immunocytochemistry

Cells were seeded at 5,000 cells/coverslip on glass coverslips (Merlan Scientific, Mississauga, ON, Canada) coated with poly-D-lysine (Millipore Sigma). Cells were cultured for 24 h (U251N and HMC3) or 48 h (primary human astrocytes) before treatment. Following treatment, cells were fixed in 4% paraformaldehyde (10 min), permeabilized with 0.1% Triton X-100 (10 min), blocked in 10% goat serum in PBS (Gibco) for 1 h and incubated with primary antibodies overnight at 4 °C: rabbit anti-GFAP (1:500, Abcam, ab7260), mouse anti-Lamp1 (1:500, Developmental Studies Hybridoma Bank, H4A3-c), rat anti-lipocalin-2 (1:500, R&D Systems, MAB1757), rabbit anti-NFkB p65 (1:500, Abcam, ab16502) or rabbit anti-phospho-STAT3 Y705 (1:500, Abcam, ab76315). Cells were washed in PBS three times and incubated with secondary antibodies for 1 h at RT: goat anti-rabbit Alexa Fluor 488 (1:1000, Thermo Fisher, Mississauga, ON, Canada, A27034), goat anti-rat Alexa Fluor 647 (1:500, Thermo Fisher, A21247) or goat anti-mouse Alexa Fluor 488 (1:1000, Thermo Fisher, A28175). Cells were washed with PBS and nuclei were labeled with Hoechst 33342 (10 μM, 10 min). After three more washings with PBS, coverslips were mounted on microscope slides (Diamed, Mississauga, ON, Canada) using Aqua-Poly/Mount. Samples were imaged using a fluorescence microscope (Leica DMI4000B, Leica) and intracellular fluorescence was analyzed in ImageJ. The nuclear and/or cytoplasmic fluorescence of NFκB, LCN2 and phosphor-STAT3 for each cell was measured and normalized to the nuclear or cytoplasmic area. The background fluorescence was subtracted.

### 2.8. Immunohistochemistry

Immunohistochemistry was performed as previously published [33]. In brief, human brain sections were dewaxed in xylene and rehydrated in ethanol. Antigen retrieval was performed in citrate buffer. Following blocking, samples were incubated with primary antibodies: rat anti-lipocalin-2 (1:500, R&D Systems, MAB1757), mouse anti-IBA1 (1:300, Invitrogen, MA5-27726) or rabbit anti-GFAP (1:250 Millipore, MAB144P) overnight at 4 °C. Samples were washed and incubated with secondary antibodies: goat anti-rat Alexa Fluor 647 (1:500, Thermo Fisher Scientific, A21247), goat anti-mouse Alexa Fluor 647 (1:300, Thermo Fisher Scientific, A28181) or goat anti-rabbit Alexa Fluor 488 (1:500, Thermo Fisher, A27034) for 1 h at RT. Nuclei were labeled with DAPI (1 µg/mL, 5 min, Molecular Probes, Ottawa, ON, Canada) and samples were mounted on microscope slides using Dako mounting medium (Dako, Burlington, ON, Canada). Samples were imaged using a fluorescence microscope. The samples were harvested under a protocol approved by the Montreal Neurological Hospital’s research ethics board (NEU-10-066). Consent was given by all patients and controls (aged 55–76). Tissues were from the cerebral cortex.

### 2.9. Lipid Droplet Imaging

Cells seeded on glass coverslips at 5,000 cells/coverslip were cultured for 24 h before treatment. Following treatment, cells were washed twice with PBS and fixed in 4% paraformaldehyde (10 min). Cells were washed with PBS and incubated with BODIPY 493/503 (10 μM, Thermo Fisher) and Hoechst 33342 (10 μM) for 10 min. Cells were washed with PBS four times, then mounted on microscope slides using EverBrite (Biotium, Burlington, ON, Canada). Samples were imaged using a fluorescence microscope.

### 2.10. MTT Assay

Following treatment, organoids were washed in PBS twice and incubated in fresh media in the presence of 3-(4,5-dimethylthiazol-2-yl)-2,5-diphenyltetrazolium bromide (MTT) (0.5 mg/mL, Millipore Sigma) for 1 h at 37  °C. The media was removed, and cells were lysed in 500 μL dimethyl sulfoxide (Santa Cruz, Dallas, TX, USA). Samples were measured at 595 nm in triplicate using a microplate reader (Spark 10M, Tecan, Männedorf, Switzerland). Measurements were normalized to the organoid weight.

### 2.11. Statistics

Statistical significance was determined using one-way ANOVA followed by the Student’s *t*-test. *p*-values lesser than 0.05 were deemed significant. The Bonferroni correction was applied for multiple comparisons.

## 3. Results

### 3.1. dPGS Are Internalized in 3D Cerebral Organoids

**Figure 1:**
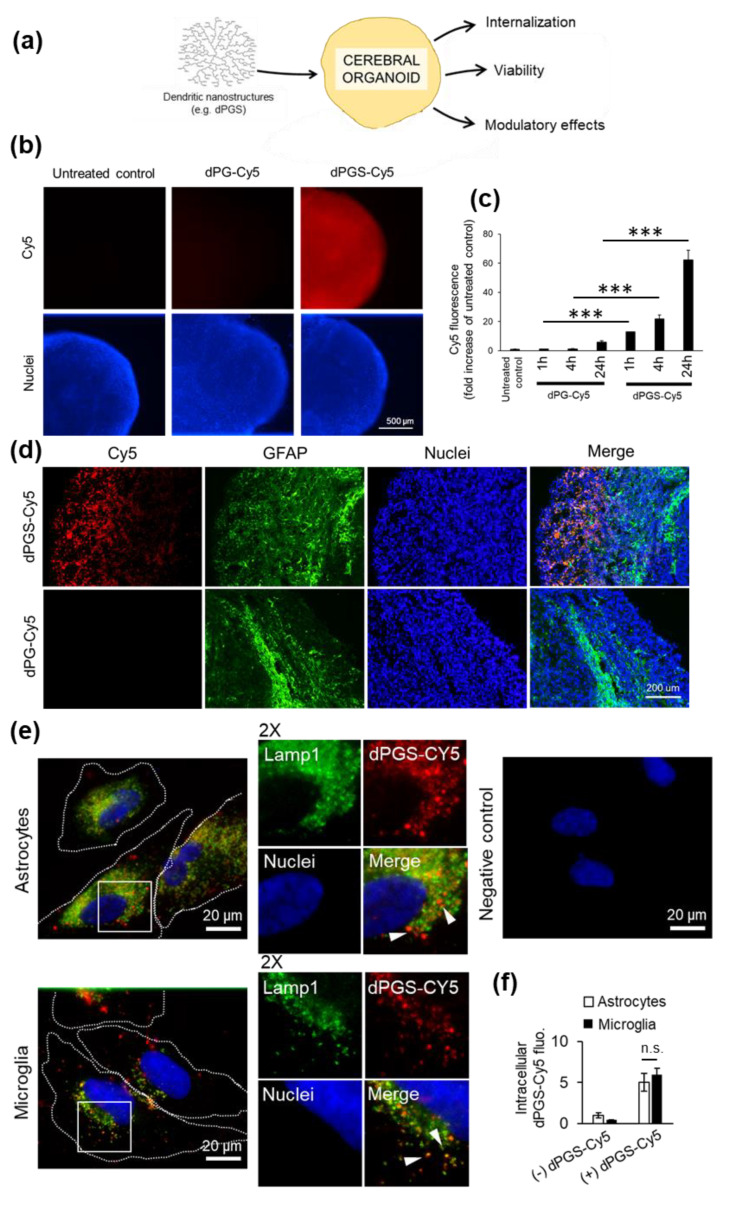
(**a**) Schematic representation of cerebral organoid usage for nanostructure screening. (**b**) Human cerebral organoids were treated with dendritic polyglycerol (dPG)-Cy5 (1 μM) or dendritic polyglycerol sulfate (dPGS)-Cy5 (1 μM) for 1 h, 4 h or 24 h. Nuclei were labeled with Hoechst 33342. Organoids were imaged using a fluorescence microscope. (**c**) Cy5 fluorescence in organoids was analyzed in ImageJ. Shown are the average fluorescence per condition ± SEM. At least 90 organoids were analyzed from three independent experiments. *** *p* < 0.001 (**d**) Fluorescence micrographs of human cerebral organoids treated with dPG-Cy5 (1 μM) or dPGS-Cy5 (1 μM) for 24 h and labeled for glial fibrillary acidic protein (GFAP). Nuclei were labeled with Hoechst 33342. (**e**) Primary human astrocytes and human HMC3 microglia internalization of dPGS-Cy5 (1 μM) after 24 h. dPGS-Cy5 (red) is partially co-localized with Lamp1-labeled lysosomal compartments (green). The negative control was prepared in the absence of dPGS-Cy5 and primary antibody to account for background fluorescence. Nuclei were labeled with Hoechst 33342 (blue). Cells were imaged using a fluorescence microscope. (**f**) Shown are the average intracellular Cy5 fluorescence per cell expressed as fold change from untreated cells ± SD. At least 90 cells were analyzed in two independent experiments. n.s. Non-significant.

Human cerebral organoids were generated from human iPSCs according to an established protocol [9]. A timeline of organoid formation and biomarkers used to delineate dynamic linage progression is illustrated in Figure S1. We used these organoids after a 100-day maturation to test dendritic polyglycerol sulfate (dPGS) as a model nanostructure with anti-inflammatory properties in human neural cells, as well as other types of emerging nanostructures with biomedical applications, such as metallic gold nanoclusters [35,36,37,38,39]. Gold nanoclusters have been shown to affect cellular stress and organellar function thanks to their unique physicochemical properties, but little work has been done in human neural cells. The nanostructures were screened for cytotoxic effects (Figure 1a and Figure S2) and results showed that they can be studied in human neural cells without jeopardizing the viability of the organoid cultures. This provides the basis for further investigations on nanomedical applications in a translationally-relevant model. The first step was to show if dPGS was internalized by neural cells within the organoids themselves. To this end, we treated organoids with fluorescently (Cy5)-labeled dPGS (Figure 1). Time-course experiments showed that dPGS-Cy5 was internalized within 1 h (12.8 ± 2.2 SEM fold increase over baseline fluorescence), then progressively more over 24 h (64.5 ± 11.4 SEM fold increase) (Figure 1b,c). Compared to dPGS, internalization of the non-sulfated dendritic polyglycerol (dPG) was low (8.7 ± 2.1 SEM fold increase after 24 h) (Figure 1b,c), underlining the important role of the terminal sulfate groups for cell internalization. In the current study, we showed that GFAP-labeled astrocytes within the outermost layer (200–300 μm) of the cerebral organoids contain abundant amounts of dPGS (Figure 1d).

### 3.2. dPGS Internalization in Human Microglia and Normal Astrocytes

Given the importance of glial cells in regulating inflammatory processes in the brain, we further studied the intracellular effects of dPGS in astrocytes and particularly microglia, which are absent from the cerebral organoids. We established in cell monolayer cultures that human microglia and astrocytes internalized comparable amounts of dPGS within 24 h of treatment (Figure 1e,f). Fluorescence imaging at the single cell level showed that dPGS-Cy5 accumulated in the perinuclear area (Figure 1e), and was partially co-localized with lysosomal compartments labeled with Lamp1 [40]. Given that microglia activated by pro-inflammagens (e.g., LPS) are associated with increased sequestration of neutral lipids into lipid droplets (LDs) [41,42] (Figure S3b), we investigated if dPGS could have modulatory effects on lysosomes, which interact with LDs through lipophagy and other processes [43,44]. This was suggested by changes in lysosomal positioning, with an increase in perinuclear lysosomes (Figure S3a). In turn, treatment with dPGS prevented LPS-induced lipid droplet accumulation (Figure S3b).

### 3.3. dPGS Reduced Microglia-Stimulated Lipocalin-2 in Cerebral Organoid Models

**Figure 2:**
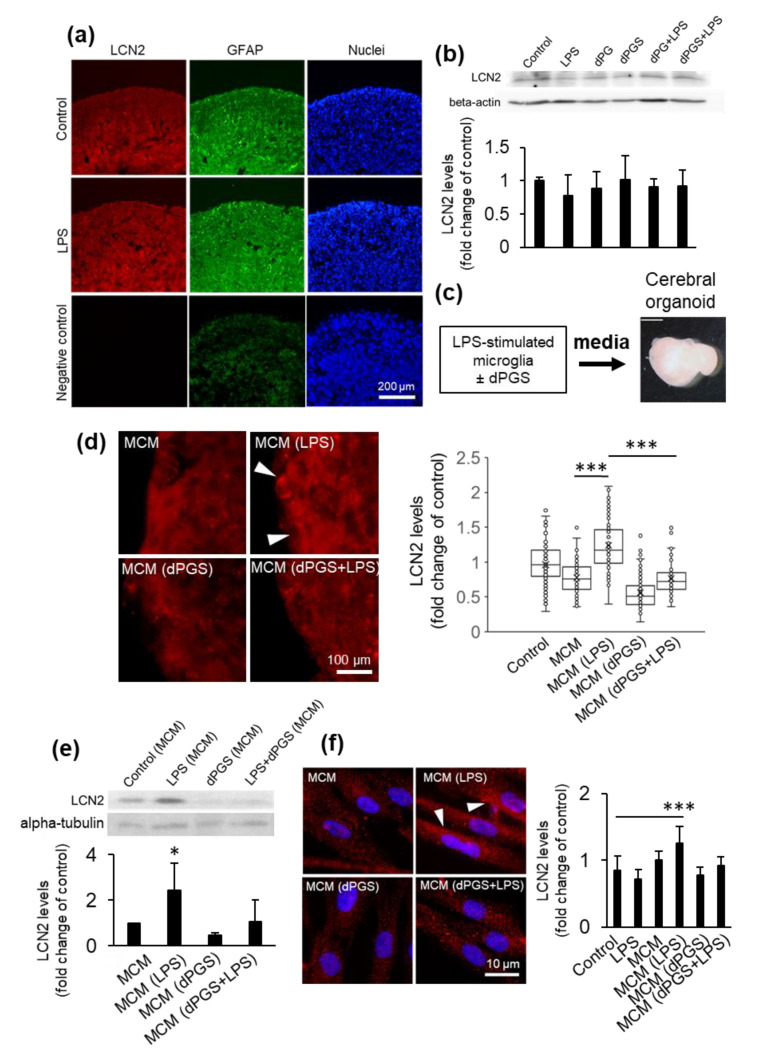
Lipocalin-2 levels in human cerebral organoids. (**a**) Micrographs of lipocalin-2 (LCN2) detected by immunofluorescence in organoid cryosections following treatment with LPS (10 ng/mL) for 24 h. Astrocytes were labeled with GFAP and nuclei with Hoechst 33342. The negative control was prepared in the absence of primary antibodies to account for background fluorescence. Samples were imaged using a fluorescence microscope. (**b**) LCN2 levels in organoids treated with lipopolysaccharide (LPS) (10 ng/mL) with or without dPG (1 μM) and dPGS (1 μM) for 24 h and measured by Western blot, with beta-actin as loading control. Quantification shown are the average intracellular LCN2 levels ±SD in organoids based on immunofluorescence images shown in (**a**). A total of 27 samples were analyzed from three independent experiments. (**c**) Schematic representation of media conditioning from microglia used for cerebral organoid treatment. (**d**) Representative fluorescence micrographs of intracellular LCN2 levels in organoids treated with conditioned media from microglia (MCM) treated with LPS (10 ng/mL), dPGS (1 μM) for 24 h. Quantifications show the average and single-cell levels of LCN2 fluorescence in cryosections. A total of 1017 cells were analyzed from independent experiments. *** *p* < 0.001 (**e**) LCN2 levels from organoids treated as in (**d**) and measured by Western blot, with alpha-tubulin as loading control. Shown are the average LCN2 levels from two independent experiments. * *p* < 0.05 (**f**) Fluorescence micrographs of LCN2 abundance in primary human astrocytes treated as in (**d**) and measured using immunocytochemistry. Shown are the average intracellular LCN2 levels in astrocytes as fold increase of the untreated control. A total of 477 cells from two independent experiments were analyzed. *** *p* < 0.001.

Considering the importance of microglia-astrocyte crosstalk in rodent [19] and human neural cells [1,3], we measured LCN2 abundance in human neural cells in response to LPS, a prototypical pro-inflammagen whose levels are exacerbated in endotoxemia and sepsis. The presence of microglia is required to stimulate the synthesis of LCN2 in the neural cells, as levels in response to LPS remained comparable to that of the untreated control in the absence of microglia from the organoids (Figure 2a,b and Figure S4). In turn, conditioned media from LPS-stimulated microglia induced a significant increase in intracellular and extracellular LCN2 in the cerebral organoid cultures (Figure 2c–e), confirming that this upregulation is microglia-dependent in human neural cells. Given that astrocytes play a significant role in inflammatory processes in the brain, we investigated if there was increased LCN2 abundance in these cells in response to microglia activation. Similarly to the results from cerebral organoids, direct stimulation of astrocytes with LPS did not have a significant effect (Figure 2f). In contrast, treatment with conditioned media from LPS-activated microglia increased LCN2 abundance, whereas the presence of dPGS returned LCN2 abundance to control levels (Figure 2f). dPGS alone and conditioned media from resting microglia did not have significant effects (Figure 2d–f). Finally, to show that dPGS is not cytotoxic to human neural cells, we measured mitochondrial metabolic activity using the MTT assay. Results from these studies showed that neither dPGS nor dPG affected mitochondrial metabolic activity in 3D organoids for up to 72 h (Figure S2). Our earlier studies indicated that in rodent cells, concentrations of dPGS up to 100 μM did not cause any notable cytotoxicity [18]. These results encouraged us to investigate dPGS as a microglia modulator and indirect suppressor of glioblastoma invasiveness.

### 3.4. Microglia and dPGS Modulate Glioblastoma Invasiveness

**Figure 3:**
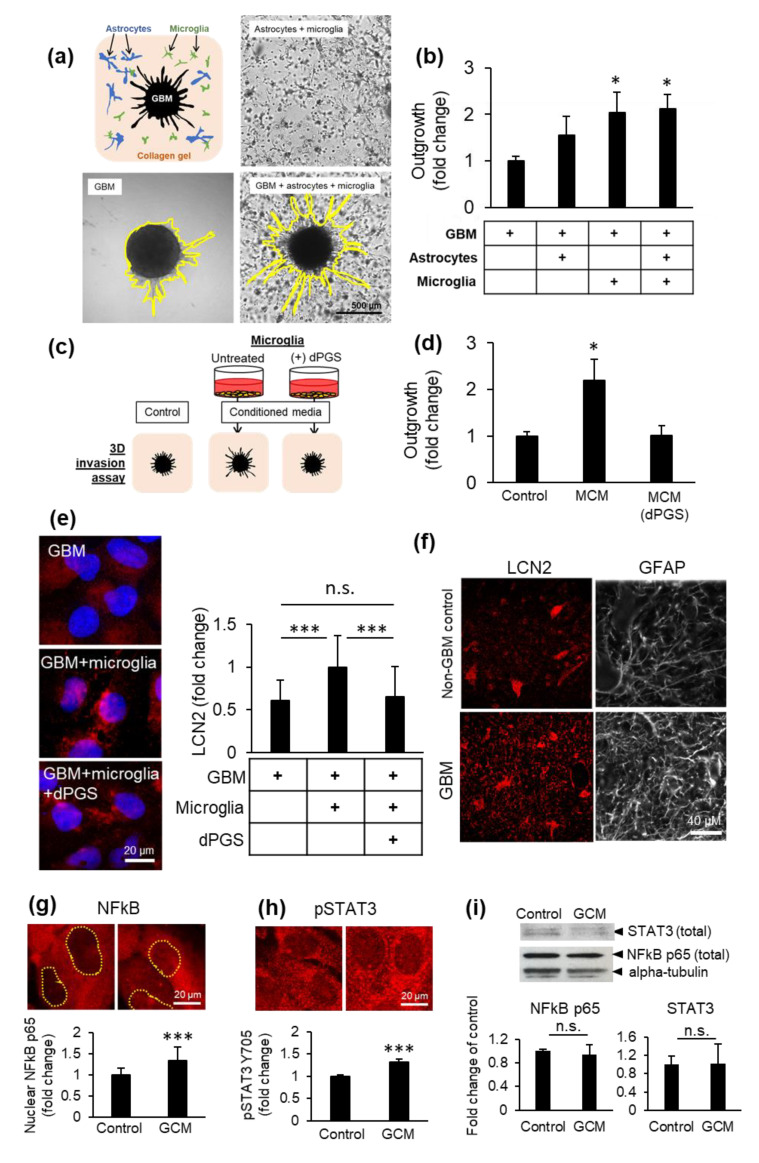
Glioblastoma (GBM) invasiveness in 3D co-cultures. (**a**) Collagen gels were seeded with human primary astrocytes and human HMC3 microglia in the presence or absence of a glioblastoma tumoroid. (**b**) Glioblastoma invasiveness in the presence or absence of microglia and astrocytes in 3D co-cultures after 6 days. Shown are the average distance of outgrowth from the tumoroid as a fold change of the tumoroid monoculture ±SEM. A total of 32 tumoroids were tested from at least three independent experiments. * *p* < 0.05 (**c**) Schematic representation of microglial conditioned media (MCM) used to treat collagen-embedded tumoroids. (**d**) Glioblastoma tumoroid outgrowth in the presence of conditioned media from microglia treated or not with dPGS (1 μM) after 6 days ±SEM. A total of 29 tumoroids were tested from at least four independent experiments. * *p* < 0.05 (**e**) Representative fluorescence micrographs showing intracellular LCN2 (red) in glioblastoma cells in the presence or absence of microglia cells in direct co-culture and dPGS (1 μM) for 24 h. LCN2 was fluorescently immunolabeled and cells were imaged using a fluorescence microscope. Quantifications show the average intracellular LCN2 levels ± SD per cell. At least 500 cells were analyzed from five independent experiments. *** *p* < 0.001; n.s. Non-significant. (**f**) Fluorescence micrographs of LCN2 (red) and GFAP (white) in human brain sections from non-cancerous brain or glioblastoma tumor tissues. LCN2 and GFAP were fluorescently labeled by immunohistochemistry and imaged using a fluorescence microscope. (**g,h**) Activation of transcription factors NFκB and STAT3 in microglia in response to glioblastoma secreted factors. Human HMC3 microglia were treated with glioblastoma conditioned media (GCM) for 24 h, after which NFκB p65 and phosphorylated STAT3 Tyr705 were fluorescently immunolabeled (red) and cells were imaged using a fluorescence microscope. Shown are (g) the average nuclear NFκB p65 level per cell ± SEM (237 cells from three independent experiments) and (h) the average pSTAT3 Y705 level per cell ± SEM (317 cells from three independent experiments) *** *p* < 0.001. (**i**) Total NFκB p65 and STAT3 protein abundance in microglia treated as in (g,h). Protein levels were determined by measurements of immunopositive bands in Western blots. Alpha-tubulin was used as loading control. Shown are the average protein abundance of NFκB p65 and STAT3 from three independent experiments. n.s. Non-significant.

Glioblastoma multiforme is an aggressive brain tumor of astrocytic origin for which complete resection is often impossible due to its invasive nature [29,45]. To model the impact of microglia and astrocytes on glioblastoma invasiveness, we designed a 3D invasion assay examining the propensity of the cancer cells to migrate into the surrounding collagen matrix. Human microglia and primary human astrocytes were embedded into the collagen matrix, wherein a tumoroid was then implanted (Figure 3a). Over time, the normal neural cells extend processes while cancer cells migrate radially from the tumoroid. Glioblastoma outgrowth from the tumoroid into the surrounding collagen was facilitated by the presence of microglia and astrocytes (Figure 3a,b), an effect replicated using microglia-conditioned media and inhibited by dPGS (Figure 3c,d). This indicates that neural cells secrete factors (extracellular matrix degradation enzymes, cytokines, growth factors) that can stimulate tumor invasiveness [46,47,48]. In contrast, tumoroids grown in isolation or within cerebral organoids in the absence of microglia had comparatively less outgrowth over time (Figure 3a,b and Figure S5), and normal human astrocytes migrated in a scattered pattern without radial outgrowth (Figure S6).

To characterize the effects of glioblastoma on its surrounding immune cells, glioblastoma cells were directly co-cultured with microglia cells and showed significantly higher LCN2 levels compared to glioblastoma cells in monoculture (Figure 3e). High LCN2 and GFAP levels were also observed in tumor tissues from glioblastoma patients as compared to control brain tissues (Figure 3f). To investigate the mechanism behind LCN2 upregulation, microglia were stimulated with glioblastoma-conditioned media. This resulted in increased nuclear NFκB and STAT3 phosphorylation, two key transcription factors for immune activation (Figure 3g,h) that are also hyperactivated in glioblastoma. The protein levels of total NFκB and STAT3 in the cells remained comparable to the control (Figure 3i).

**Figure 4:**
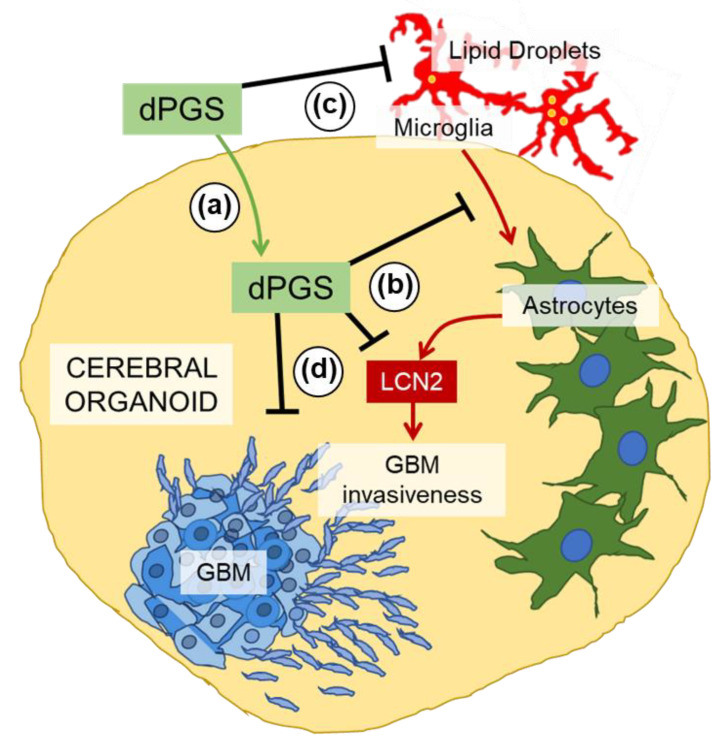
Schematic representation of the proposed modulatory effects of dPGS on microglia, astrocyte and glioblastoma crosstalk. (**a**) dPGS is internalized in cerebral organoids, and (**b**) downregulates LCN2 produced by microglia-induced astrocytes, thereby (**c**) reducing markers of inflammation (e.g., lipid droplets) and (**d**) glioblastoma invasiveness.

On the other hand, dPGS was internalized into glioblastoma tumoroids (Figure S7) and decreased LCN2 levels in glioblastoma cells in vitro (Figure 3e). This demonstrates proof of concept for using dPGS as a modulator of the tumor microenvironment (Figure 4).

## 4. Discussion

The key question addressed in this study is: can dendritic polyglycerol sulfates with intrinsic anti-inflammatory properties reduce inflammatory markers in cerebral organoids and glioblastoma invasiveness by modulating microglia activity? Mechanistic studies in 3D human neural cultures with new drugs or nanostructures are sparse due to the limited availability of neural tissues. To overcome such a problem, several protocols have been developed for the generation of 3D cerebral organoids and other types of organoids [4,5,7,9]. Human cerebral organoids derived from iPSCs showing properties of different neural cell types are valuable models to study the interactions and effects of nanostructures in 3D. One of the most advanced organoids containing vasculature resembling that of the brain was recently proposed to investigate physiology and pathological changes in neurological disorders [49]. Although our organoids are not as complex as Cakir’s, we took advantage of cerebral organoids to reveal how microglia exposed to the pro-inflammagen LPS and the dendritic nanostructure dPGS affect astrocyte reactivity. We show clear anti-inflammatory effects from dPGS and provide the first evidence for this in human brain cells. There are still several limitations to overcome: (1) the integration of resident and peripheral immune cells recruited chemotactically and (2) the contribution of endothelial cells in vasculature as part of the blood–brain barrier.

Our results in organoid cultures showed that the presence of sulfate groups on dPGS was critical for the internalization of nanostructures, as it allowed binding to selectin receptors [17,19,22]. Intracellular changes associated with dPGS included an increase in perinuclear lysosomes, which typically have lower luminal pH and increased degradation activity [44,50], functions often impaired in aging and neurodegenerative diseases. This is interesting given the role of lysosomes in processing LDs, which are upregulated in inflammation and cancer [41,42]. LDs are promiscuous organelles: depending on the cell type, cellular context and their intracellular location, they can play a protective role or become damaging to neural and other cells if peroxidized lipids are released. In aggressive tumors (e.g., glioblastoma and breast), they provide energy for cancer cells to thrive and can sequester lipophilic anticancer agents, reducing the rate at which they reach their desirable targets [51,52,53]. Their numbers are highly upregulated in glioblastoma compared to normal astrocytes (Figure S3c,d), and their inhibition was shown to enhance the effectiveness of pharmacological agents [51].

As now shown in human neural cells, LCN2 can be produced by microglia-stimulated astrocytes. Microglia themselves are not significant contributors to LCN2 upregulation due to the LCN2 gene being suppressed by SRSF3 [54]. Instead, they secrete other cytokines (e.g., IL-6, TNFα) and alarmins under pro-inflammatory conditions that can activate astrocytes and other neural cells [54,55]. Several of these were shown to bind heparan sulfate [23,56], suggesting that dPGS prevented microglia-mediated LCN2 production by blocking some soluble factors and preventing downstream signaling (Figure 2d,e). One of the candidate cytokines is interleukin-6 released from microglia, to which dPGS was shown to bind in our earlier studies using surface plasmon resonance [19].

Cytokines and alarmins play key roles in the tumor microenvironment [57,58,59,60,61]. The cancer cells secrete factors that actively recruit microglia and peripheral macrophages to the tumor site. In turn, these immune cells produce cytokine and growth factors that can promote cancer progression [32]. In this context, microglia-astrocyte crosstalk forms a positive feedback loop that maintains an unfavorable tumor microenvironment [30,62]. LCN2 is an acute-phase protein with emerging roles in the brain and in cancer. It was shown to complex with and enhance the activity of metalloproteinase-9, a protease secreted by cancer cells that breaks down the extracellular matrix and promotes invasiveness [63,64]. This is significant given that dPGS can downregulate LCN2 in neural cells and decrease glioblastoma invasiveness. dPGS can bind P-selectin, which is expressed in the tumor endothelium and in glioblastoma cells [65]. As dPGS can also serve as a nanocarrier, it has the potential to deliver anti-cancer agents [65,66]. Nanocarriers such as functionalized dendrimers for siRNA and drug delivery [67,68,69] merit testing in human organoid models to reveal how combination therapy could affect interplay between cancer cells and the tumor microenvironment. The modulatory effect of dPGS on organelles could impact glioblastoma cells in several ways: (1) the increase in perinuclear lysosomes could decrease lysosomal exocytosis and the release of extracellular matrix-degrading enzymes (e.g., cathepsins) [70,71], (2) a reduction in lipid droplet size and number [72] is an indicator of reduced microglia hyperactivity, thus fewer cytokines and trophic factors contributing to glioblastoma invasiveness and (3) a decrease in lipid droplets prevents the sequestration of lipophilic anti-cancer agents, allowing them to reach their intracellular targets (e.g., curcumin) [51].

## 5. Conclusions

Our results show that dPGS is an attractive candidate as an anti-inflammatory polyglycerol dendrimer able to modulate human microglia-astrocyte crosstalk. This dendrimer regulated lipocalin 2 abundance in human neural organoids. In the context of inflammation associated with glioblastoma multiforme, dPGS limited glioblastoma invasiveness by modulating microglial activation. Overall, these studies propose the evaluation of well-defined nanostructures in well-characterized human organoids and co-cultures. Cerebral organoids merit further studies as complementary 3D models in nanoscience, but they require rigorous characterization and application of standardized procedures to be widely used [73]. Such three-dimensional systems together with co-cultures and in vivo experiments will provide a valuable evaluation platform for the internalization, cytotoxicity and modulatory effects of nanotherapeutics in human neural cells.

## Supplementary Materials

The following are available online at https://www.mdpi.com/2073-4409/9/11/2434/s1, Figure S1: Maturation and characterization of cerebral organoids, Figure S2: Mitochondrial metabolic activity in cerebral organoids following treatment with nanostructures, Figure S3: Changes in lysosomes and lipid droplets in activated microglia, Figure S4: Absence of microglia in the cerebral organoids, Figure S5: Glioblastoma tumoroid invasiveness in cerebral organoids, Figure S6: Movement of normal human astrocytes in 3D culture, Figure S7: Internalization of fluorescent dPGS-Cy5 in glioblastoma tumoroids.

## References

[1] Paolicelli R.C., Bergamini G., Rajendran L. (2019). Cell-to-cell Communication by Extracellular Vesicles: Focus on Microglia. Neuroscience.

[2] Simon E., Obst J., Gomez-Nicola D. (2019). The Evolving Dialogue of Microglia and Neurons in Alzheimer’s Disease: Microglia as Necessary Transducers of Pathology. Neuroscience.

[3] Pósfai B., Cserép C., Orsolits B., Dénes Á. (2019). New Insights into Microglia–Neuron Interactions: A Neuron’s Perspective. Neuroscience.

[4] Rossi G., Manfrin A., Lutolf M.P. (2018). Progress and potential in organoid research. Nat. Rev. Genet..

[5] Li M., Izpisua Belmonte J.C. (2019). Organoids—Preclinical Models of Human Disease. N. Engl. J. Med..

[6] Takebe T., Wells J.M. (2019). Organoids by design. Science.

[7] Marshall J.J., Mason J.O. (2019). Mouse vs man: Organoid models of brain development & disease. Brain Res..

[8] Breschi A., Gingeras T.R., Guigó R. (2017). Comparative transcriptomics in human and mouse. Nat. Rev. Genet..

[9] Lancaster M.A., Knoblich J.A. (2014). Generation of cerebral organoids from human pluripotent stem cells. Nat. Protoc..

[10] Chen X., Rocha C., Rao T., Durcan T.M. (2019). NeuroEDDU protocols_iPSC culture. Zenodo.

[11] Ioannidis J.P.A., Kim B.Y.S., Trounson A. (2018). How to design preclinical studies in nanomedicine and cell therapy to maximize the prospects of clinical translation. Nat. Biomed. Eng..

[12] Aparicio-Blanco J., Martín-Sabroso C., Torres-Suárez A.-I. (2016). In vitro screening of nanomedicines through the blood brain barrier: A critical review. Biomaterials.

[13] Jackson S., Meeks C., Vézina A., Robey R.W., Tanner K., Gottesman M.M. (2019). Model systems for studying the blood-brain barrier: Applications and challenges. Biomaterials.

[14] Akinc A., Maier M.A., Manoharan M., Fitzgerald K., Jayaraman M., Barros S., Ansell S., Du X., Hope M.J., Madden T.D. (2019). The Onpattro story and the clinical translation of nanomedicines containing nucleic acid-based drugs. Nat. Nanotechnol..

[15] Havel H., Finch G., Strode P., Wolfgang M., Zale S., Bobe I., Youssoufian H., Peterson M., Liu M. (2016). Nanomedicines: From Bench to Bedside and Beyond. AAPS J..

[16] Hrkach J., Hoff D.V., Ali M.M., Andrianova E., Auer J., Campbell T., Witt D.D., Figa M., Figueiredo M., Horhota A. (2012). Preclinical Development and Clinical Translation of a PSMA-Targeted Docetaxel Nanoparticle with a Differentiated Pharmacological Profile. Sci. Transl. Med..

[17] Rades N., Licha K., Haag R. (2018). Dendritic Polyglycerol Sulfate for Therapy and Diagnostics. Polymers (Basel).

[18] Maysinger D., Groger D., Lake A., Licha K., Weinhart M., Chang P.K.-Y., Mulvey R., Haag R., McKinney R.A. (2015). Dendritic Polyglycerol Sulfate Inhibits Microglial Activation and Reduces Hippocampal CA1 Dendritic Spine Morphology Deficits. Biomacromolecules.

[19] Maysinger D., Lalancette-Hébert M., Ji J., Jabbour K., Dernedde J., Silberreis K., Haag R., Kriz J. (2019). Dendritic polyglycerols are modulators of microglia-astrocyte crosstalk. Future Neurol..

[20] Budde H., Sorns M.-S., Welker P., Licha K., Wolff H., Riggert J., Wulf G., Legler T.J. (2016). Dendritic polyglycerol sulfate attenuates murine graft-versus-host disease. Ann. Hematol..

[21] Xu X., Ballauff M. (2019). Interaction of Lysozyme with a Dendritic Polyelectrolyte: Quantitative Analysis of the Free Energy of Binding and Comparison to Molecular Dynamics Simulations. J. Phys. Chem. B.

[22] Dernedde J., Rausch A., Weinhart M., Enders S., Tauber R., Licha K., Schirner M., Zügel U., von Bonin A., Haag R. (2010). Dendritic polyglycerol sulfates as multivalent inhibitors of inflammation. PNAS.

[23] Parish C.R. (2006). The role of heparan sulphate in inflammation. Nat. Rev. Immunol..

[24] Türk H., Haag R., Alban S. (2004). Dendritic Polyglycerol Sulfates as New Heparin Analogues and Potent Inhibitors of the Complement System. Bioconjugate Chem..

[25] Maysinger D., Ji J., Moquin A., Hossain S., Hancock M.A., Zhang I., Chang P.K.Y., Rigby M., Anthonisen M., Grutter P. (2018). Dendritic Polyglycerol Sulfates in the Prevention of Synaptic Loss and Mechanism of Action on Glia. ACS Chem. Neurosci..

[26] Prinz M., Jung S., Priller J. (2019). Microglia Biology: One Century of Evolving Concepts. Cell.

[27] Masuda T., Sankowski R., Staszewski O., Prinz M. (2020). Microglia Heterogeneity in the Single-Cell Era. Cell Rep..

[28] Rodríguez-Gómez J.A., Kavanagh E., Engskog-Vlachos P., Engskog M.K.R., Herrera A.J., Espinosa-Oliva A.M., Joseph B., Hajji N., Venero J.L., Burguillos M.A. (2020). Microglia: Agents of the CNS Pro-Inflammatory Response. Cells.

[29] Li C., Wang S., Yan J.-L., Piper R.J., Liu H., Torheim T., Kim H., Zou J., Boonzaier N.R., Sinha R. (2019). Intratumoral Heterogeneity of Glioblastoma Infiltration Revealed by Joint Histogram Analysis of Diffusion Tensor Imaging. Neurosurgery.

[30] Broekman M.L., Maas S.L.N., Abels E.R., Mempel T.R., Krichevsky A.M., Breakefield X.O. (2018). Multidimensional communication in the microenvirons of glioblastoma. Nat. Rev. Neurol..

[31] Chen Z., Hambardzumyan D. (2018). Immune Microenvironment in Glioblastoma Subtypes. Front. Immunol..

[32] Anfray C., Ummarino A., Andón F.T., Allavena P. (2020). Current Strategies to Target Tumor-Associated-Macrophages to Improve Anti-Tumor Immune Responses. Cells.

[33] Zhang I., Beus M., Stochaj U., Le P.U., Zorc B., Rajic Z., Petrecca K., Maysinger D. (2018). Inhibition of glioblastoma cell proliferation, invasion, and mechanism of action of a novel hydroxamic acid hybrid molecule. Cell Death Discov..

[34] Del Duca D., Werbowetski T., Del Maestro R.F. (2004). Spheroid preparation from hanging drops: Characterization of a model of brain tumor invasion. J. Neurooncol..

[35] Ji J., Moquin A., Bertorelle F., Ky Chang P., Antoine R., Luo J., McKinney R.A., Maysinger D. (2019). Organotypic and primary neural cultures as models to assess effects of different gold nanostructures on glia and neurons. Nanotoxicology.

[36] Bonačić-Koutecký V., Antoine R. (2019). Enhanced two-photon absorption of ligated silver and gold nanoclusters: Theoretical and experimental assessments. Nanoscale.

[37] Maysinger D., Gran E.R., Bertorelle F., Fakhouri H., Antoine R., Kaul E.S., Samhadaneh D.A., Stochaj U. (2019). Gold nanoclusters elicit homeostatic perturbations in glioblastoma cells and adaptive changes of lysosomes. Theranostics.

[38] Hakkinen H. (2008). Atomic and electronic structure of gold clusters: Understanding flakes, cages and superatoms from simple concepts. Chem. Soc. Rev..

[39] Jin R., Zeng C., Zhou M., Chen Y. (2016). Atomically Precise Colloidal Metal Nanoclusters and Nanoparticles: Fundamentals and Opportunities. Chem. Rev..

[40] Macairan J.-R., Zhang I., Clermont-Paquette A., Naccache R., Maysinger D. (2020). Optical Sensing: Ratiometric pH Sensing in Living Cells Using Carbon Dots (Part. Part. Syst. Charact. 1/2020). Part. Part. Syst. Charact..

[41] Olzmann J.A., Carvalho P. (2019). Dynamics and functions of lipid droplets. Nat. Rev. Mol. Cell Biol..

[42] Marschallinger J., Iram T., Zardeneta M., Lee S.E., Lehallier B., Haney M.S., Pluvinage J.V., Mathur V., Hahn O., Morgens D.W. (2020). Lipid-droplet-accumulating microglia represent a dysfunctional and proinflammatory state in the aging brain. Nat. Neurosci..

[43] Schulze R.J., Sathyanarayan A., Mashek D.G. (2017). Breaking fat: The regulation and mechanisms of lipophagy. Biochim. Biophys Acta.

[44] Pu J., Guardia C.M., Keren-Kaplan T., Bonifacino J.S. (2016). Mechanisms and functions of lysosome positioning. J. Cell Sci..

[45] Davis M.E. (2016). Glioblastoma: Overview of Disease and Treatment. Clin. J. Oncol. Nurs..

[46] Ramachandran R.K., Sørensen M.D., Aaberg-Jessen C., Hermansen S.K., Kristensen B.W. (2017). Expression and prognostic impact of matrix metalloproteinase-2 (MMP-2) in astrocytomas. PLoS ONE.

[47] Vollmann-Zwerenz A., Leidgens V., Feliciello G., Klein C.A., Hau P. (2020). Tumor Cell Invasion in Glioblastoma. Int. J. Mol. Sci..

[48] Medema J.P. (2013). Cancer stem cells: The challenges ahead. Nat. Cell Biol..

[49] Cakir B., Xiang Y., Tanaka Y., Kural M.H., Parent M., Kang Y.-J., Chapeton K., Patterson B., Yuan Y., He C.-S. (2019). Engineering of human brain organoids with a functional vascular-like system. Nat. Methods.

[50] Johnson D.E., Ostrowski P., Jaumouillé V., Grinstein S. (2016). The position of lysosomes within the cell determines their luminal pH. J. Cell Biol..

[51] Zhang I., Cui Y., Amiri A., Ding Y., Campbell R.E., Maysinger D. (2016). Pharmacological inhibition of lipid droplet formation enhances the effectiveness of curcumin in glioblastoma. Eur. J. Pharm. Biopharm..

[52] Dubey R., Stivala C.E., Nguyen H.Q., Goo Y.-H., Paul A., Carette J.E., Trost B.M., Rohatgi R. (2020). Lipid droplets can promote drug accumulation and activation. Nat. Chem. Biol..

[53] Treyer A., Mateus A., Wiśniewski J.R., Boriss H., Matsson P., Artursson P. (2018). Intracellular Drug Bioavailability: Effect of Neutral Lipids and Phospholipids. Mol. Pharm..

[54] Boutej H., Rahimian R., Thammisetty S.S., Béland L.-C., Lalancette-Hébert M., Kriz J. (2017). Diverging mRNA and Protein Networks in Activated Microglia Reveal SRSF3 Suppresses Translation of Highly Upregulated Innate Immune Transcripts. Cell Rep..

[55] Liddelow S.A., Guttenplan K.A., Clarke L.E., Bennett F.C., Bohlen C.J., Schirmer L., Bennett M.L., Münch A.E., Chung W.-S., Peterson T.C. (2017). Neurotoxic reactive astrocytes are induced by activated microglia. Nature.

[56] Mummery R.S., Rider C.C. (2000). Characterization of the heparin-binding properties of IL-6. J. Immunol..

[57] Roesch S., Rapp C., Dettling S., Herold-Mende C. (2018). When Immune Cells Turn Bad—Tumor-Associated Microglia/Macrophages in Glioma. Int. J. Mol. Sci..

[58] Sims G.P., Rowe D.C., Rietdijk S.T., Herbst R., Coyle A.J. (2010). HMGB1 and RAGE in Inflammation and Cancer. Annu. Rev. Immunol..

[59] Rapoport B.L., Steel H.C., Theron A.J., Heyman L., Smit T., Ramdas Y., Anderson R. (2020). High Mobility Group Box 1 in Human Cancer. Cells.

[60] Matarredona E.R., Pastor A.M. (2020). Extracellular Vesicle-Mediated Communication between the Glioblastoma and Its Microenvironment. Cells.

[61] Conti I., Varano G., Simioni C., Laface I., Milani D., Rimondi E., Neri L.M. (2020). miRNAs as Influencers of Cell–Cell Communication in Tumor Microenvironment. Cells.

[62] Gieryng A., Pszczolkowska D., Walentynowicz K.A., Rajan W.D., Kaminska B. (2017). Immune microenvironment of gliomas. Lab. Invest..

[63] Kobara H., Miyamoto T., Suzuki A., Asaka R., Yamada Y., Ishikawa K., Kikuchi N., Ohira S., Shiozawa T. (2013). Lipocalin2 enhances the matrix metalloproteinase-9 activity and invasion of extravillous trophoblasts under hypoxia. Placenta.

[64] Lin Y., Ren J., Qu X. (2014). Catalytically active nanomaterials: A promising candidate for artificial enzymes. Acc. Chem. Res..

[65] Ferber S., Tiram G., Sousa-Herves A., Eldar-Boock A., Krivitsky A., Scomparin A., Yeini E., Ofek P., Ben-Shushan D., Vossen L.I. (2017). Co-targeting the tumor endothelium and P-selectin-expressing glioblastoma cells leads to a remarkable therapeutic outcome. Elife.

[66] Sousa-Herves A., Würfel P., Wegner N., Khandare J., Licha K., Haag R., Welker P., Calderón M. (2015). Dendritic polyglycerol sulfate as a novel platform for paclitaxel delivery: Pitfalls of ester linkage. Nanoscale.

[67] Mendes L.P., Sarisozen C., Luther E., Pan J., Torchilin V.P. (2019). Surface-engineered polyethyleneimine-modified liposomes as novel carrier of siRNA and chemotherapeutics for combination treatment of drug-resistant cancers. Drug Deliv..

[68] Subhan M.A., Torchilin V.P. (2019). Efficient nanocarriers of siRNA therapeutics for cancer treatment. Transl Res..

[69] Gerecke C., Edlich A., Giulbudagian M., Schumacher F., Zhang N., Said A., Yealland G., Lohan S.B., Neumann F., Meinke M.C. (2017). Biocompatibility and characterization of polyglycerol-based thermoresponsive nanogels designed as novel drug-delivery systems and their intracellular localization in keratinocytes. Nanotoxicology.

[70] Machado E., White-Gilbertson S., van de Vlekkert D., Janke L., Moshiach S., Campos Y., Finkelstein D., Gomero E., Mosca R., Qiu X. (2015). Regulated lysosomal exocytosis mediates cancer progression. Sci. Adv..

[71] Sundler R. (1997). Lysosomal and cytosolic pH as regulators of exocytosis in mouse macrophages. Acta Physiol. Scand..

[72] Kepsutlu B., Wycisk V., Achazi K., Kapishnikov S., Pérez-Berná A.J., Guttmann P., Cossmer A., Pereiro E., Ewers H., Ballauff M. (2020). Cells Undergo Major Changes in the Quantity of Cytoplasmic Organelles after Uptake of Gold Nanoparticles with Biologically Relevant Surface Coatings. ACS Nano.

[73] Marx V. (2020). Reality check for organoids in neuroscience. Nat. Methods.

