# Supplementary material for: Nanotherapeutic Modulation of Human Neural Cells and Glioblastoma in Organoids and Monocultures
Source: Cells. 2020 Nov 7;9(11):2434. doi: 10.3390/cells9112434 (PMC7695149; doi:10.3390/cells9112434)
Supplement: Supplementary file 1 [file cells-09-02434-s001.pdf]

Supplemental materials

Nanotherapeutic modulation of human neural cells and glioblastoma in organoids and monocultures

Issan Zhang, Paula Lépine, Chanshuai Han, María Lacalle-Aurioles, Carol X.-Q. Chen, Rainer Haag, Thomas M. Durcan and Dusica Maysinger

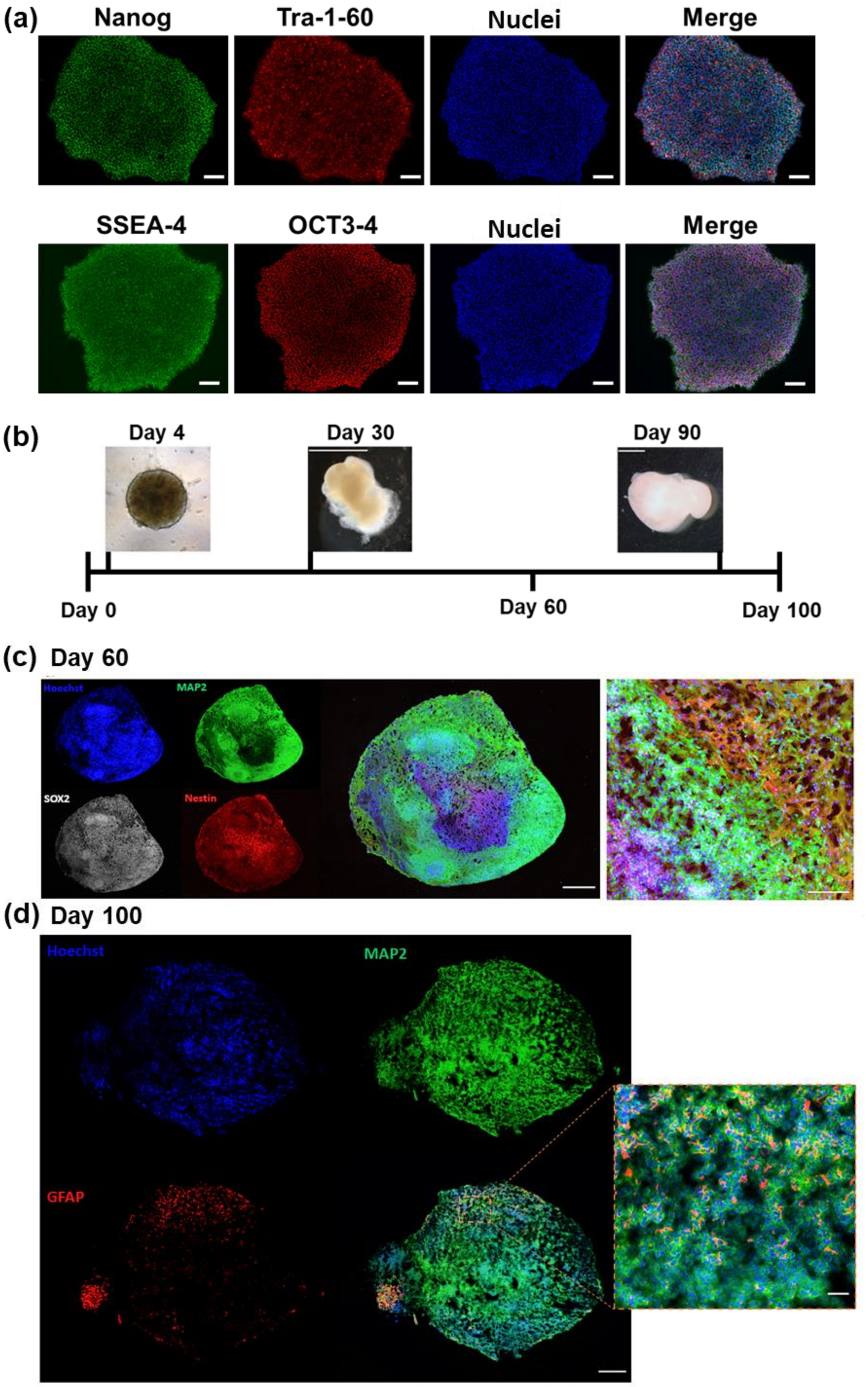

Figure S1. (a) Expression of pluripotency markers in human iPSCs. Immunofluorescence images of NCRM1 cells labelled for the pluripotency markers SSEA-4, Oct3-4, Nanog and Tra-1-60 with nuclear staining (DAPI). Scale bar: 200 µm. **(b)** Timeline of cerebral organoid growth and development from iPSCs, over 100 days. **(c,d)** Stemness (SOX2, Nestin) and neural markers in cerebral organoids at days 60 and 100. Immunofluorescence staining for mature neurons expressing microtubule associated protein 2 (MAP2) and astrocytes expressing glial fibrillary acidic protein (GFAP). Staining of cell nuclei with Hoechst 33342. Scale bar: 200 µm (for higher magnification: scale bar: 25 µm).

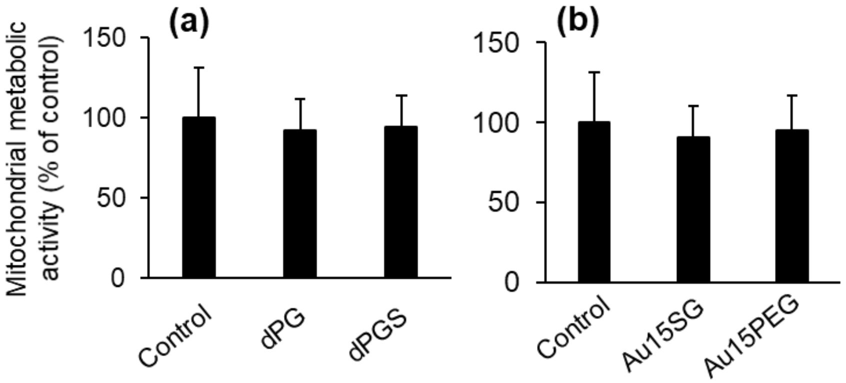

Figure S2. (a) Mitochondrial metabolic activity in cerebral organoids following treatment with dPG and dPGS (1 µM, 72 h), using the MTT assay. Shown are the average percentage mitochondrial metabolic activity ±SD. **(b)** Mitochondrial metabolic activity in cerebral organoids following treatment with gold nanoclusters Au15SG and Au15PEG (1 µM, 72 h), using the MTT assay. Shown are the average percentage mitochondrial metabolic activity ±SD.

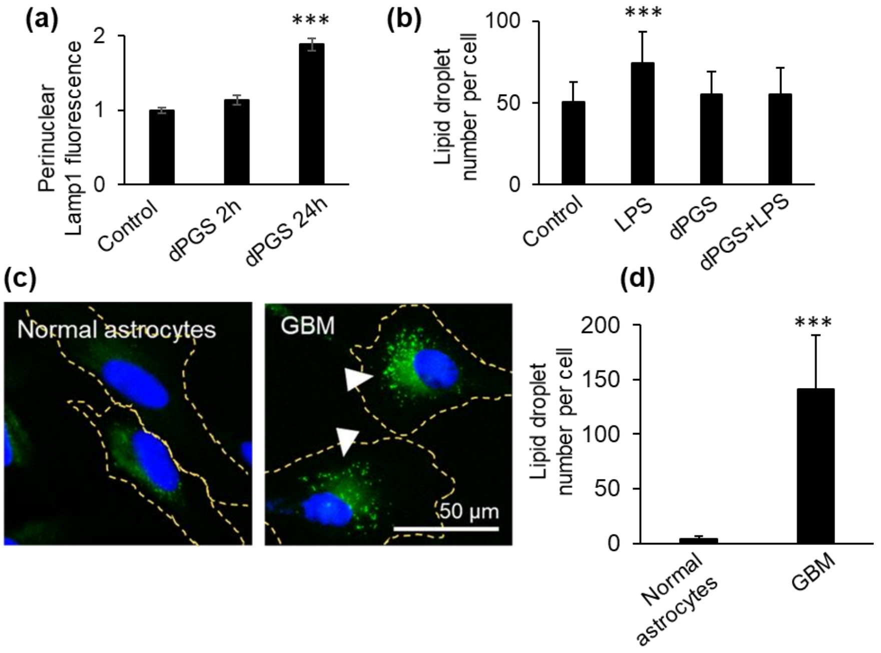

Figure S3. (a) Increase in microglial perinuclear lysosomal marker Lamp1 in response to dPGS treatment. Human HMC3 microglia were treated with dPGS (1 µM) for 2 h or 24 h, then Lamp1 was fluorescently labeled by immunocytochemistry. Cells were imaged using a fluorescent microscope and perinuclear signal (within 5 µm of the nucleus) was measured in ImageJ. Shown are the average perinuclear Lamp1 signal per cell ±SEM. 356 cells were analyzed from two independent experiments. ***$p<0.001$ **(b)** Increase in microglial lipid droplet numbers in response to LPS and normalization of lipid droplet numbers with dPGS. Human HMC3 microglia were treated with LPS (10 ng/mL) with or without dPGS (1 µM) for 24 h, after which lipid droplets were fluorescently labeled with BODIPY 493/503 and imaged using a fluorescence microscope. The number of lipid droplets per cell was counted manually. Shown are the average number of lipid droplets per cell ±SEM. At least 250 cells from three independent experiments were analyzed. ***$p<0.001$ **(c)** Fluorescence micrographs of lipid droplets in human primary astrocytes and U251N glioblastoma (astrocytoma) cells. Lipid droplets were labelled as in (b) and imaged using a fluorescence microscope. **(d)** Shown are the average number of lipid droplets in human primary astrocytes and glioblastoma cells ±SD. 51 cells were analyzed from two independent experiments. ***$p<0.001$

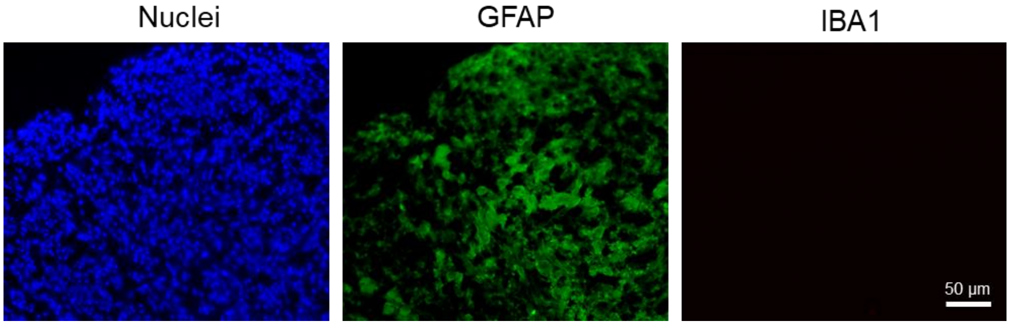

Figure S4. Absence of microglia from the cerebral organoids, as shown by IBA1 labeling. Immunofluorescence staining for astrocytes with GFAP and microglia with IBA1. Staining of cell nuclei with Hoechst 33342.

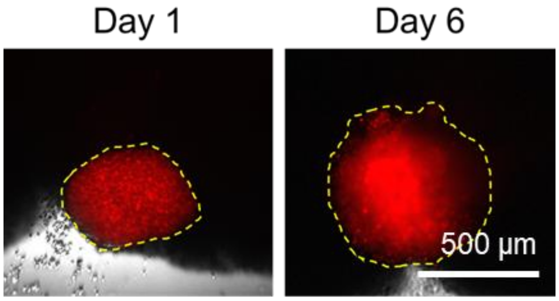

Figure S5. Glioblastoma tumoroid has limited invasiveness in cerebral organoids in the absence of microglia cells. Fluorescently-labeled glioblastoma tumoroids were implanted in mini-brains and imaged after 6 days for infiltrative invasiveness using a fluorescence microscope.

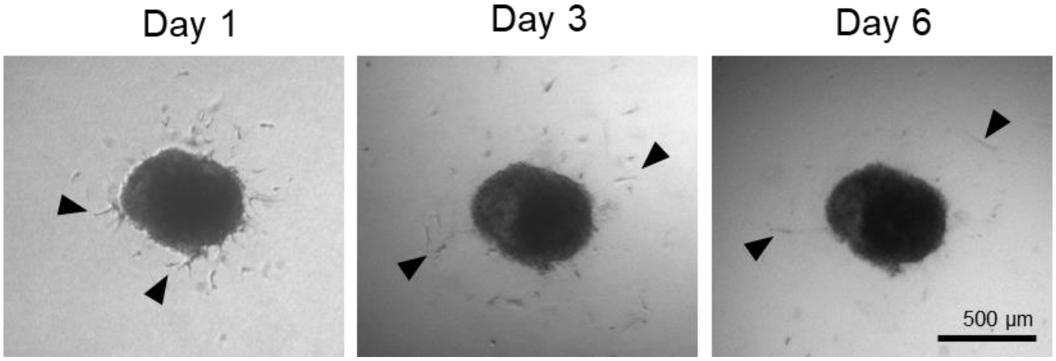

Figure S6. Movement of normal human astrocytes in 3D culture. Human astrocyte spheroids were implanted into collagen gels and cultured for 6 days. Spheroids were imaged using brightfield.

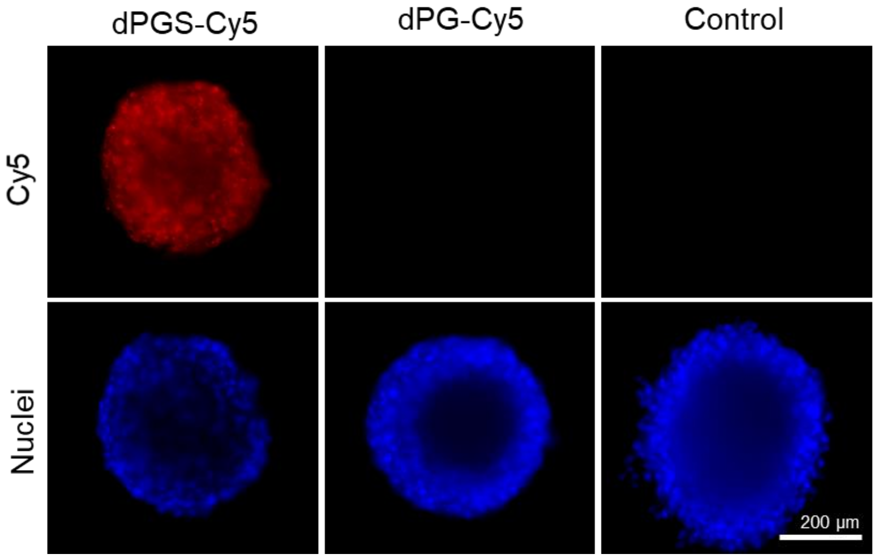

Figure S7. Internalization of fluorescent dPGS-Cy5 in glioblastoma tumoroids. Shown are representative fluorescence micrographs of glioblastoma tumoroids treated with dPGS-CY5 (red) or dPG-Cy5 for 24h at 1 µM. Nuclei were labeled with Hoechst 33342 (blue). Tumoroids were imaged using a fluorescence microscope.
